# Supplementary material for: Quantum-effective exact multiple patterns matching algorithms for biological sequences
Source: PeerJ Comput Sci. 2022 May 12;8:e957. doi: 10.7717/peerj-cs.957 (PMC9138144; doi:10.7717/peerj-cs.957)
Supplement: Supplemental Information 1 [file peerj-cs-08-957-s001.docx]

**APPENDIX A**

$\boldsymbol{List of Abbreviations}$

**Table A1 Abbreviated names used throughout the article for algorithms and other significant terms**

| $\boldsymbol{CPU, GPU \&}\boldsymbol{QPU}$ | **:** | *Central processing unit, graphics processing unit, and Quantum processing unit* |
| --- | --- | --- |
| $\boldsymbol{CRAM \&}\boldsymbol{QMEM}$ | **:** | *Classical memory or Classical-RAM, and Quantum memory or Quantum-RAM* |
| $\boldsymbol{EnQPBEA-MPM}$ | **:** | *Enhanced* $QMEM$ *processing-based exact algorithm for multiple pattern matching* |
| $\boldsymbol{EnQBCEA-MPM}$ | **:** | *Enhanced quantum-based combined exact algorithm for multiple pattern matching* |
| $\boldsymbol{DNA}\boldsymbol{\&}\boldsymbol{RNA}$ | **:** | *Deoxyribonucleic acid, and Ribonucleic acid* |
| $\boldsymbol{QEM}\boldsymbol{\& GSO}$ | **:** | *Quantum-exact match, and Grover’s search operator* |
| $\boldsymbol{KMP}\boldsymbol{\&}\boldsymbol{BM}$ | **:** | *Knuth-Morris-Pratt algorithm, and Boyer-Moore algorithm* |
| $\boldsymbol{AC, CW}\boldsymbol{\&}\boldsymbol{WM}$ | **:** | *Aho-Corasick algorithm, Commentz-Walter algorithm, and Wu-Manber algorithm* |
| $\boldsymbol{BDM}\boldsymbol{\&}\boldsymbol{BSOM}$ | **:** | *Backward DAWG Matching algorithm, and Backward Set Oracle Matching algorithm* |
| $\boldsymbol{SO, SA}\boldsymbol{\&}\boldsymbol{BNDM}$ | **:** | *Shift-OR algorithm, Shift-AND algorithm, and Backward Non-Deterministic DAWG Matching* |
| $\boldsymbol{RV-Algorithm}$ | **:** | *Ramesh-Vinay algorithm based on quantum deterministic sampling method* |
| $\boldsymbol{QEPM}\boldsymbol{\&}\boldsymbol{QAPM}$ | **:** | *Quantum exact pattern matching algorithm, and Quantum approximate pattern matching algorithm* |
| $\boldsymbol{QPBE}\boldsymbol{\&}\boldsymbol{QBCE}$ | **:** | $QMEM$ *processing-based exact algorithm, and Quantum-based combined exact algorithm* |
| $\boldsymbol{QEMP}\boldsymbol{\&}\boldsymbol{QAMP}$ | **:** | *Quantum exact multiple pattern, and Quantum approximate multiple pattern matching algorithm* |
| $\boldsymbol{QuEST}$ | **:** | *Quantum exact simulation toolkit (used for the proposed quantum algorithm simulation)* |
| $\boldsymbol{ANF}$ | **:** | *Algebraic normal form (used to realize* $QMEM$ *and other quantum specific operations using* $QuEST$*)* |
| $\boldsymbol{HD}$ | **:** | *Hamming distance method (used for approximate text filtering and matching)* |
| $\boldsymbol{QAF}$ | **:** | *Quantum approximate filtering (used for reducing the size of search space in* $EnQBCEA$ *algorithm)* |
| $\boldsymbol{EQP} \boldsymbol{\&} \boldsymbol{BQP}$ | **:** | *Exact quantum polynomial time, and Bounded error quantum polynomial time* |
| $\boldsymbol{QC}\boldsymbol{\&}\boldsymbol{QAE}$ | **:** | *Quantum counting (used for unknown number of solutions), and Quantum amplitude estimation* |
| $\boldsymbol{Exact-QC}$ | **:** | *Exact quantum counting algorithm (used to obtain accurate number of search solutions)* |
| $\boldsymbol{Approx.}\boldsymbol{-QC}$ | **:** | *Approximate quantum counting algorithm (used to estimate the number of search solutions)* |
| $\boldsymbol{SARS-CoV-2}$ | **:** | *Severe Acute Respiratory Syndrome Corona-Virus 2 (used as gene sequence database in simulation)* |
| $\boldsymbol{QASM}$ | **:** | *Quantum assembly instructions (used to record operations executed on registers by quantum gates)* |
| $\boldsymbol{No. of CIP}$ | **:** | *Number of correctly identified patterns (evaluated parameter in simulation)* |
| $\boldsymbol{No. of}\boldsymbol{I}\boldsymbol{IP}$ | **:** | *Number of incorrectly identified patterns (evaluated parameter in simulation)* |
| $\boldsymbol{No. of}\boldsymbol{IM}\boldsymbol{P}$ | **:** | *Number of incorrectly missed patterns (evaluated parameter in simulation)* |
| $\boldsymbol{Avg. ET}\boldsymbol{\& CRAM-WS}$ | **:** | *Average Execution Time of Searching, and* $CRAM$ *Workspace (evaluated parameters in simulation)* |
