# Supplementary material for: Quantum-effective exact multiple patterns matching algorithms for biological sequences
Source: PeerJ Comput Sci. 2022 May 12;8:e957. doi: 10.7717/peerj-cs.957 (PMC9138144; doi:10.7717/peerj-cs.957)
Supplement: Supplemental Information 2 [file peerj-cs-08-957-s002.docx]

**APPENDIX B**

$\boldsymbol{Nomenclatures used in Proposed Algorithms}$**:** $\boldsymbol{EnQPBEA \& EnQBCEA}$

**Table B1 Used symbols in our quantum algorithms with precise descriptions**

| $\boldsymbol{\vert}\boldsymbol{\psi}_{\boldsymbol{qubits}}\boldsymbol{\rangle}$ | **:** | *Quantum state of superposition, with the qubits to exploring qubits-dimensional Hilbert Space as* $2^{qubits}$*amplitudes with all column vectors* |
| --- | --- | --- |
| ${\boldsymbol{\vert}\boldsymbol{T}_{\boldsymbol{2}^{\boldsymbol{n}}\boldsymbol{\times w}}\boldsymbol{\rangle}}_{\boldsymbol{QMEM}}$ | **:** | *Superposition based quantum memory* $QMEM$ *with* $N=2^{n}$ *addresses, each of word size* $w$*, access by n-qubits address* ${\vert T_{n}\rangle}_{QA}$*, w-qubits data register* ${\vert T_{[w]}\rangle}_{QD}$ |
| ${\boldsymbol{\vert}\boldsymbol{T}_{\boldsymbol{i}}\boldsymbol{\rangle}}_{\boldsymbol{QA}}$ | **:** | $i^{th}$ *index remain in superposition of text indices of size* ${n=log}_{2} N$ *qubits or* ${tq=log}_{2} t$ *(filtered)* |
| ${\boldsymbol{\vert}\boldsymbol{T}_{\boldsymbol{[i]}}\boldsymbol{\rangle}}_{\boldsymbol{QD}}$ | **:** | $i^{th}$ *index data corresponds to entangled address of pattern size* $w=M*{log}_{2} \left\vert\Sigma\right\vert$ *qubits* |
| ${\boldsymbol{\vert}\boldsymbol{P}\boldsymbol{\rangle}}_{\boldsymbol{DR}}$ | **:** | *Pattern data register to store* $M$ *length pattern of size* $M$ $*$ ${log}_{2} \left\vert\Sigma\right\vert$ *qubits in separate manner* |
| $\boldsymbol{\vert wait}\boldsymbol{\rangle}$***,*** $\boldsymbol{\vert left}\boldsymbol{\rangle}$***,*** $\boldsymbol{\vert right}\boldsymbol{\rangle}$ | **:** | *Qutrit switches which remains in superposition* $\vert wait\rangle$*, and transform to states as* $\vert left\rangle$ *or* $\vert right\rangle$ |
| $\boldsymbol{\vert q}\boldsymbol{\rangle}$ | **:** | *Ancillary qubit initialize to* $\vert-\rangle$ *used in phase inversion to mark amplitude by picking phase factor* $-1$ |
| $\boldsymbol{\vert}\boldsymbol{q}_{\boldsymbol{Comp}}\boldsymbol{\rangle}$ | **:** | *Single qubit whose value is returned to function as the outcome of Boolean oracle of exact search* |
| $\boldsymbol{\vert}\boldsymbol{same}_{\boldsymbol{[qubits]}}\boldsymbol{\rangle}$ | **:** | *This keeps the state of quantum register as with same instance by using the explored qubits* |
| $\boldsymbol{\vert}\boldsymbol{zeroes}_{\boldsymbol{qubits}}\boldsymbol{\rangle}$ | **:** | *It initializes the quantum register with zero values as per specified qubits within the register* |
| ${\boldsymbol{\vert}\boldsymbol{T}_{\boldsymbol{n+1}}\boldsymbol{\rangle}}_{\boldsymbol{AX}}$ | **:** | *An auxiliary register of text size with additional ancillary qubit used for approximate text filtering* |
| $\boldsymbol{LA}\left[ \boldsymbol{\ldots} \right]$ | **:** | *Location array to classically store the* $t$ *filtered text indices as per the measured outcome of filtering* |
| ${\boldsymbol{\vert}\boldsymbol{T}_{\boldsymbol{tq}}\boldsymbol{\rangle}}_{\boldsymbol{QL}}$ | **:** | *A location register of size* ${tq=log}_{2} t$ *qubits to access filtered text indices stored in array* $LA\left[ t \right]$ |
| $\boldsymbol{U}_{\boldsymbol{QMEM}}$ | **:** | *A unitary transformation to load data of size* ${\vert T_{[w]}\rangle}_{QD}$ *as* ${\vert T_{[i]}\rangle}_{QD}$ *for all entangled address* ${\vert T_{i}\rangle}_{QA}$ |
| $\boldsymbol{U}_{\boldsymbol{Swap}}$ | **:** | *A unitary operator to transform qutrit from* $\vert wait\rangle$ *to* $\vert left\rangle$ *or* $\vert right\rangle$ *state,* ${U^{\dagger}}_{Swap}$ *is reverse of it.* |
| $\boldsymbol{U}_{\boldsymbol{Load}}$ | **:** | *A unitary to explore and trace the path as followed by qutrit switches, and copies the data at* ${\vert T_{[i]}\rangle}_{QD}$ *by performing round trip of bus qubits, corresponding to each address* ${\vert T_{i}\rangle}_{QA}$ *in superposition* |
| $\boldsymbol{U}_{\boldsymbol{Comp}}$ | **:** | *A unitary operation used for exact match between pattern and text substring at each index* ${\vert T_{i}\rangle}_{QA}$ |
| $\boldsymbol{U}_{\boldsymbol{mark}}$ | **:** | *A unitary operator that yields to the phase inversion of the marked solution index by using ancilla qubit* |
| $\boldsymbol{U}_{\boldsymbol{Diff}}$ | **:** | *A unitary operator for amplitude purification, that inverts probability amplitude around mean value* |
| $\boldsymbol{U}_{\boldsymbol{GetL}}$ | **:** | *A unitary transformation that gets n-qubits actual index by* ${\vert T_{i}\rangle}_{QL}$ *i.e. the memory content of* $LA\left[ t \right]$ *then store that address for its further access through* ${\vert T_{i}\rangle}_{QA}$ *i.e. address register* |
