# Supplementary material for: Quantum-effective exact multiple patterns matching algorithms for biological sequences
Source: PeerJ Comput Sci. 2022 May 12;8:e957. doi: 10.7717/peerj-cs.957 (PMC9138144; doi:10.7717/peerj-cs.957)
Supplement: Supplemental Information 4 [file peerj-cs-08-957-s004.docx]

**APPENDIX D**

$\boldsymbol{Correctness Proof of Proposed Algorithm 2}$**:** $\boldsymbol{EnQBCEA-MPM}$

***Trace Note – D1:*** *Trace steps Eqs. (24) – (31) justifies* $\boldsymbol{Quantum Approximate Filtering}$ *that identifies possible start / filtered location of each pattern*$\boldsymbol{P}_{\boldsymbol{k}}\boldsymbol{\in}\boldsymbol{P}$ *using* $\boldsymbol{c}^{\boldsymbol{th}}$ *quantum core* $\boldsymbol{QCore}_{\boldsymbol{c}}$ *accessing* $\boldsymbol{T}$ *on shared*$\boldsymbol{QMEM}$***.*** *Iterate these number of steps until all likely* $\boldsymbol{t}_{\boldsymbol{k}}$ *indices corresponding to* $\boldsymbol{P}_{\boldsymbol{k}}$ *be not stored in*$\boldsymbol{LA}_{\boldsymbol{k}}\left[ \boldsymbol{\ldots} \right]$***.*** *Further, trace steps Eqs. (32) – (39) describes the* *mathematical proof of correctness of* $\boldsymbol{EnQBCEA-MPM}$ *to ensure pattern match****.***

1. *Prepare each* $\boldsymbol{P}_{\boldsymbol{k}}\boldsymbol{\in}\boldsymbol{P}$ *of size* $\boldsymbol{w}\boldsymbol{=}\boldsymbol{M}_{\boldsymbol{k}}\boldsymbol{\times}\boldsymbol{log}_{\boldsymbol{2}} \left| \boldsymbol{\Sigma} \right|$ *in register* ${\boldsymbol{|}\boldsymbol{P}\boldsymbol{\rangle}}_{\boldsymbol{DRk}}$ *of* $\boldsymbol{c}^{\boldsymbol{th}}$ *quantum core* $\boldsymbol{QCore}_{\boldsymbol{c}}$ *and then store* ${\boldsymbol{|}\boldsymbol{i}_{\boldsymbol{j}}\boldsymbol{\rangle}}_{\boldsymbol{k}}$ *in array* $\boldsymbol{SL}_{\boldsymbol{k}}\left[ \boldsymbol{M} \right]$ *as preprocessed marking of possible start locations of distinct symbols for each pattern contained in register* ${\boldsymbol{|}\boldsymbol{P}\boldsymbol{\rangle}}_{\boldsymbol{DRk}}$

${\boldsymbol{|}\boldsymbol{P}_{\left[ \boldsymbol{0 to}\boldsymbol{M}_{\boldsymbol{k}}\boldsymbol{-1} \right]}\boldsymbol{\rangle}}_{\boldsymbol{DRk}}\boldsymbol{\leftarrow}\left\{ \boldsymbol{P}_{\boldsymbol{1}}\boldsymbol{,.,}\boldsymbol{P}_{\boldsymbol{k}}\boldsymbol{,}\boldsymbol{.,}\boldsymbol{P}_{\boldsymbol{m}} \right\}$ **&** $\boldsymbol{SL}_{\boldsymbol{k}}\left[ \boldsymbol{M} \right]\boldsymbol{\leftarrow Distinct Symbols}\left( \boldsymbol{P}_{\boldsymbol{k}} \right)$ **(24)**

1. *Prepare quantum registers of each* $\boldsymbol{c}^{\boldsymbol{th}}$ *quantum core* $\boldsymbol{QCore}_{\boldsymbol{c}}$ *in zero states including address and auxiliary registers, and the pattern register is still kept initialized*

${{\boldsymbol{|}\boldsymbol{\psi}_{\boldsymbol{n}}\boldsymbol{\rangle}}_{\boldsymbol{k}}}^{\boldsymbol{0}}$ **:** ${{\boldsymbol{|}\boldsymbol{T}_{\boldsymbol{0}}\boldsymbol{\rangle}}_{\boldsymbol{QAk}}}^{\boldsymbol{\otimes n}} \boldsymbol{\bigotimes}{{\boldsymbol{|}\boldsymbol{T}_{\boldsymbol{[0]}}\boldsymbol{\rangle}}_{\boldsymbol{QDk}}}^{\boldsymbol{\otimes w}}\boldsymbol{\bigotimes}{{\boldsymbol{|}\boldsymbol{T}_{\boldsymbol{0}}\boldsymbol{\rangle}}_{\boldsymbol{AXk}}}^{\boldsymbol{\otimes(n+1)}}$ $\boldsymbol{\bigotimes}$ ${\boldsymbol{|}\boldsymbol{P}_{\left[ \boldsymbol{0 to}\boldsymbol{M}_{\boldsymbol{k}}\boldsymbol{-1} \right]}\boldsymbol{\rangle}}_{\boldsymbol{DRk}}$ **(25)**

1. *Initialize superposition state in address register & entangle auxiliary register of* $\boldsymbol{QCore}_{\boldsymbol{c}}$ *as*

${{\boldsymbol{|}\boldsymbol{\psi}_{\boldsymbol{n}}\boldsymbol{\rangle}}_{\boldsymbol{k}}}^{\boldsymbol{1}}$ **:** $\boldsymbol{H}^{\boldsymbol{\otimes n}}\left( \boldsymbol{I}^{\boldsymbol{\otimes n}} \boldsymbol{I}^{\boldsymbol{\otimes w}}\boldsymbol{\bigotimes}\boldsymbol{I}^{\boldsymbol{\otimes(n+1)}} \right) {{\boldsymbol{|}\boldsymbol{\psi}_{\boldsymbol{n}}\boldsymbol{\rangle}}_{\boldsymbol{k}}}^{\boldsymbol{0}}$

**:** $\frac{\boldsymbol{1}}{\sqrt{\boldsymbol{N}}} \sum_{\boldsymbol{i=0}}^{\boldsymbol{N- 1}} {\boldsymbol{|}\boldsymbol{T}_{\boldsymbol{i}}\boldsymbol{\rangle}}_{\boldsymbol{QAk}} \boldsymbol{\bigotimes}{\boldsymbol{|}\boldsymbol{T}_{\boldsymbol{[0]}}\boldsymbol{\rangle}}_{\boldsymbol{QDk}} \boldsymbol{\bigotimes}{\boldsymbol{|}\boldsymbol{T}_{\boldsymbol{0}}\boldsymbol{\rangle}}_{\boldsymbol{AXk}} \boldsymbol{\bigotimes} {\boldsymbol{|}\boldsymbol{P}\boldsymbol{\rangle}}_{\boldsymbol{DRk}}$ **(26)**

1. *For all* ${\boldsymbol{|}\boldsymbol{T}_{\boldsymbol{i}}\boldsymbol{\rangle}}_{\boldsymbol{QAk}}$ *in their separate uniform quantum superposition state* ${{\boldsymbol{|}\boldsymbol{\psi}_{\boldsymbol{n}}\boldsymbol{\rangle}}_{\boldsymbol{k}}}^{\boldsymbol{1}}$***,*** *load data at* ${\boldsymbol{|}\boldsymbol{T}_{\boldsymbol{[i]}}\boldsymbol{\rangle}}_{\boldsymbol{QDk}}$ *as per addresses* ${\boldsymbol{|}\boldsymbol{T}_{\boldsymbol{i}}\boldsymbol{\rangle}}_{\boldsymbol{QAk}}$ *by applying* $\boldsymbol{QMEM Transformation}$ *of Eq. (1) as*

$\boldsymbol{QMEM Transformation}\boldsymbol{\leftarrow}\left( \boldsymbol{U}_{\boldsymbol{QMEM}}\boldsymbol{\leftarrow}\left( {\boldsymbol{U}^{\boldsymbol{\dagger}}}_{\boldsymbol{Swap}}\left( \boldsymbol{U}_{\boldsymbol{Load}} \left( \boldsymbol{U}_{\boldsymbol{Swap}} \right) \right) \right) \right)$

*Unitary* $\boldsymbol{U}_{\boldsymbol{Load}}$ *of Eq. (3) is directly applied for data transformation from memory to register*

${{\boldsymbol{|}\boldsymbol{\psi}_{\boldsymbol{n}}\boldsymbol{\rangle}}_{\boldsymbol{k}}}^{\boldsymbol{2}}$ **:** $\boldsymbol{U}_{\boldsymbol{Load}}\left( {{\boldsymbol{|}\boldsymbol{\psi}_{\boldsymbol{n}}\boldsymbol{\rangle}}_{\boldsymbol{k}}}^{\boldsymbol{1}} \right)$

**:**$\boldsymbol{U}_{\boldsymbol{Load}}\left( {\boldsymbol{|}\boldsymbol{T}_{\boldsymbol{i}}\boldsymbol{\rangle}}_{\boldsymbol{QAk}}\boldsymbol{\bigotimes}{\boldsymbol{|}\boldsymbol{T}_{\boldsymbol{[0]}}\boldsymbol{\rangle}}_{\boldsymbol{QDk}} \right)\boldsymbol{=}\boldsymbol{U}_{\boldsymbol{Load}}\left( {\boldsymbol{|}\boldsymbol{T}_{\boldsymbol{i}}\boldsymbol{\rangle}}_{\boldsymbol{QAk}}\boldsymbol{\bigotimes}{\boldsymbol{|}\boldsymbol{T}_{\left[ \boldsymbol{0}\bigoplus\boldsymbol{i} \right]}\boldsymbol{\rangle}}_{\boldsymbol{QDk}} \right)$

**:** $\frac{\boldsymbol{1}}{\sqrt{\boldsymbol{N}}} \sum_{\boldsymbol{i=0}}^{\boldsymbol{N- 1}} {\boldsymbol{|}\boldsymbol{T}_{\boldsymbol{i}}\boldsymbol{\rangle}}_{\boldsymbol{QAk}} \boldsymbol{\bigotimes}{\boldsymbol{|}\boldsymbol{T}_{\boldsymbol{[i]}}\boldsymbol{\rangle}}_{\boldsymbol{QDk}} \boldsymbol{\bigotimes}{\boldsymbol{|}\boldsymbol{T}_{\boldsymbol{0}}\boldsymbol{\rangle}}_{\boldsymbol{AXk}}\boldsymbol{\bigotimes} {\boldsymbol{|}\boldsymbol{P}\boldsymbol{\rangle}}_{\boldsymbol{DRk}}$ **(27)**

1. *Apply unitary operator* $\boldsymbol{U}_{\boldsymbol{kSLoc}}$ *on* ${{\boldsymbol{|}\boldsymbol{\psi}_{\boldsymbol{n}}\boldsymbol{\rangle}}_{\boldsymbol{k}}}^{\boldsymbol{2}}$ *at* $\boldsymbol{QCore}_{\boldsymbol{c}}$ *to mark the occurrence of each distinct symbols of pattern as* ${\boldsymbol{|}\boldsymbol{i}_{\boldsymbol{j}}\boldsymbol{\rangle}}_{\boldsymbol{k}}$ *in the entangled auxiliary register* ${\boldsymbol{|}\boldsymbol{T}_{\boldsymbol{i}_{\boldsymbol{j}}}\boldsymbol{\rangle}}_{\boldsymbol{AXk}}$

${{\boldsymbol{|}\boldsymbol{\psi}_{\boldsymbol{n}}\boldsymbol{\rangle}}_{\boldsymbol{k}}}^{\boldsymbol{3}}$ **:** $\boldsymbol{U}_{\boldsymbol{kSLoc}}\left( {{\boldsymbol{|}\boldsymbol{\psi}_{\boldsymbol{n}}\boldsymbol{\rangle}}_{\boldsymbol{k}}}^{\boldsymbol{2}} \right)$

**:** $\boldsymbol{U}_{\boldsymbol{SLoc}}\left( \frac{\boldsymbol{1}}{\sqrt{\boldsymbol{N}}} \sum_{\boldsymbol{i=0}}^{\boldsymbol{N- 1}} {\boldsymbol{|}\boldsymbol{T}_{\boldsymbol{i}}\boldsymbol{\rangle}}_{\boldsymbol{QAk}} \boldsymbol{\bigotimes}{\boldsymbol{|}\boldsymbol{T}_{\boldsymbol{[i]}}\boldsymbol{\rangle}}_{\boldsymbol{QDk}} \boldsymbol{\bigotimes}{\boldsymbol{|}\boldsymbol{T}_{\boldsymbol{0}}\boldsymbol{\rangle}}_{\boldsymbol{AXk}}\boldsymbol{\bigotimes} {\boldsymbol{|}\boldsymbol{P}\boldsymbol{\rangle}}_{\boldsymbol{DRk}} \right)$

**:** $\frac{\boldsymbol{1}}{\sqrt{\boldsymbol{N}}} \sum_{\boldsymbol{i=0}}^{\boldsymbol{N- 1}} {\boldsymbol{|}\boldsymbol{T}_{\boldsymbol{i}}\boldsymbol{\rangle}}_{\boldsymbol{QAk}} \boldsymbol{\bigotimes}{\boldsymbol{|}\boldsymbol{T}_{\boldsymbol{[i]}}\boldsymbol{\rangle}}_{\boldsymbol{QDk}} \boldsymbol{\bigotimes}{\boldsymbol{|}\boldsymbol{T}_{\boldsymbol{i}_{\boldsymbol{j}}}\boldsymbol{\rangle}}_{\boldsymbol{AXk}}\boldsymbol{\bigotimes} {\boldsymbol{|}\boldsymbol{P}\boldsymbol{\rangle}}_{\boldsymbol{DRk}}$ **(28)**

1. *Apply unitary operator* $\boldsymbol{U}_{\boldsymbol{kPLoc}}$ *on* ${{\boldsymbol{|}\boldsymbol{\psi}_{\boldsymbol{n}}\boldsymbol{\rangle}}_{\boldsymbol{k}}}^{\boldsymbol{3}}$ *at* $\boldsymbol{QCore}_{\boldsymbol{c}}$ *to get possible start location of* ${\boldsymbol{|}\boldsymbol{P}\boldsymbol{\rangle}}_{\boldsymbol{DRk}}$ *in auxiliary register* ${\boldsymbol{|}\boldsymbol{T}_{\boldsymbol{n+1}}\boldsymbol{\rangle}}_{\boldsymbol{AXk}}$ *with index position as* $\boldsymbol{|}\boldsymbol{i-}\boldsymbol{i}_{\boldsymbol{j}}\boldsymbol{\rangle}$

${{\boldsymbol{|}\boldsymbol{\psi}_{\boldsymbol{n}}\boldsymbol{\rangle}}_{\boldsymbol{k}}}^{\boldsymbol{4}}$ **:** $\boldsymbol{U}_{\boldsymbol{kPLoc}}\left( {{\boldsymbol{|}\boldsymbol{\psi}_{\boldsymbol{n}}\boldsymbol{\rangle}}_{\boldsymbol{k}}}^{\boldsymbol{3}} \right)$

**:** $\boldsymbol{U}_{\boldsymbol{kPLoc}}\left( \frac{\boldsymbol{1}}{\sqrt{\boldsymbol{N}}} \sum_{\boldsymbol{i=0}}^{\boldsymbol{N- 1}} {\boldsymbol{|}\boldsymbol{T}_{\boldsymbol{i}}\boldsymbol{\rangle}}_{\boldsymbol{QAk}} \boldsymbol{\bigotimes}{\boldsymbol{|}\boldsymbol{T}_{\boldsymbol{[i]}}\boldsymbol{\rangle}}_{\boldsymbol{QDk}} \boldsymbol{\bigotimes}{\boldsymbol{|}\boldsymbol{T}_{\boldsymbol{i}_{\boldsymbol{j}}}\boldsymbol{\rangle}}_{\boldsymbol{AXk}}\boldsymbol{\bigotimes} {\boldsymbol{|}\boldsymbol{P}\boldsymbol{\rangle}}_{\boldsymbol{DRk}} \right)$

**:** $\frac{\boldsymbol{1}}{\sqrt{\boldsymbol{N}}} \sum_{\boldsymbol{i=0}}^{\boldsymbol{N- 1}} {\boldsymbol{|}\boldsymbol{T}_{\boldsymbol{i}}\boldsymbol{\rangle}}_{\boldsymbol{QAk}} \boldsymbol{\bigotimes}{\boldsymbol{|}\boldsymbol{T}_{\boldsymbol{[i]}}\boldsymbol{\rangle}}_{\boldsymbol{QDk}} \boldsymbol{\bigotimes}{\boldsymbol{|}\boldsymbol{T}_{\boldsymbol{i}}\boldsymbol{-}\boldsymbol{T}_{\boldsymbol{i}_{\boldsymbol{j}}}\boldsymbol{\rangle}}_{\boldsymbol{AXk}}\boldsymbol{\bigotimes} {\boldsymbol{|}\boldsymbol{P}\boldsymbol{\rangle}}_{\boldsymbol{DRk}}$ **(29)**

1. *Apply Hamming Distance at* ${\boldsymbol{|}\boldsymbol{T}_{\boldsymbol{i}}\boldsymbol{-}\boldsymbol{T}_{\boldsymbol{i}_{\boldsymbol{j}}}\boldsymbol{\rangle}}_{\boldsymbol{AXk}}$ *to check for distance between text identified substring and pattern, such that,* $\boldsymbol{HD}\boldsymbol{\leq}$ ***threshold* (30)**
2. *Perform Hadamard operation at* ${\boldsymbol{|}\boldsymbol{T}_{\boldsymbol{i}}\boldsymbol{\rangle}}_{\boldsymbol{QAk}}$ *to zeroed its indices amplitudes and then merge amplitudes of entangled indices of* ${\boldsymbol{|}\boldsymbol{T}_{\boldsymbol{i}}\boldsymbol{-}\boldsymbol{T}_{\boldsymbol{i}_{\boldsymbol{j}}}\boldsymbol{\rangle}}_{\boldsymbol{AXk}}$ *as*

${{\boldsymbol{|}\boldsymbol{\psi}_{\boldsymbol{n}}\boldsymbol{\rangle}}_{\boldsymbol{k}}}^{\boldsymbol{5}}$ **:** $\boldsymbol{H}^{\boldsymbol{\otimes n}}\left( {{\boldsymbol{|}\boldsymbol{\psi}_{\boldsymbol{n}}\boldsymbol{\rangle}}_{\boldsymbol{k}}}^{\boldsymbol{4}} \right)$

**:** $\boldsymbol{H}^{\boldsymbol{\otimes n}}\left( \frac{\boldsymbol{1}}{\sqrt{\boldsymbol{N}}} \sum_{\boldsymbol{i=0}}^{\boldsymbol{N- 1}} \boldsymbol{\alpha}_{\boldsymbol{i}} {\boldsymbol{|}\boldsymbol{T}_{\boldsymbol{i}}\boldsymbol{\rangle}}_{\boldsymbol{QAk}} \boldsymbol{\bigotimes}{\boldsymbol{|}\boldsymbol{T}_{\boldsymbol{[i]}}\boldsymbol{\rangle}}_{\boldsymbol{QDk}} {\boldsymbol{\bigotimes}\boldsymbol{\alpha}}_{\boldsymbol{i-}\boldsymbol{i}_{\boldsymbol{j}}} {\boldsymbol{|}\boldsymbol{T}_{\boldsymbol{i}}\boldsymbol{-}\boldsymbol{T}_{\boldsymbol{i}_{\boldsymbol{j}}}\boldsymbol{\rangle}}_{\boldsymbol{AXk}}\boldsymbol{\bigotimes} {\boldsymbol{|}\boldsymbol{P}\boldsymbol{\rangle}}_{\boldsymbol{DRk}} \right)$

**:** $\frac{\boldsymbol{1}}{\sqrt{\boldsymbol{N}}} \sum_{\boldsymbol{i=0}}^{\boldsymbol{N- 1}} \boldsymbol{\alpha}_{\boldsymbol{i}} {\boldsymbol{|}\boldsymbol{T}_{\boldsymbol{0}}\boldsymbol{\rangle}}_{\boldsymbol{QAk}} {\boldsymbol{\bigotimes|}\boldsymbol{T}_{\boldsymbol{[i]}}\boldsymbol{\rangle}}_{\boldsymbol{QDk}} {\boldsymbol{\bigotimes}\boldsymbol{\alpha}}_{\boldsymbol{i-}\boldsymbol{i}_{\boldsymbol{j}}} {\boldsymbol{|}\boldsymbol{T}_{\boldsymbol{i}}\boldsymbol{-}\boldsymbol{T}_{\boldsymbol{i}_{\boldsymbol{j}}}\boldsymbol{\rangle}}_{\boldsymbol{AXk}}\boldsymbol{\bigotimes} {\boldsymbol{|}\boldsymbol{P}\boldsymbol{\rangle}}_{\boldsymbol{DRk}}$ **(31)**

1. *Measure auxiliary* ${\boldsymbol{|}\boldsymbol{T}_{\boldsymbol{i}}\boldsymbol{-}\boldsymbol{T}_{\boldsymbol{i}_{\boldsymbol{j}}}\boldsymbol{\rangle}}_{\boldsymbol{AXk}}$ *and store the identified index as* $\boldsymbol{|}\boldsymbol{T}_{\boldsymbol{i}}\boldsymbol{\rangle}$ *in*$\boldsymbol{LA}_{\boldsymbol{k}}\left[ \boldsymbol{\ldots} \right]$***.***

***Trace Note – D2:*** *As measurement destroys quantum state, so in each call at* $\boldsymbol{c}^{\boldsymbol{th}}$ *quantum core* $\boldsymbol{QCore}_{\boldsymbol{c}}$ *on behalf of*$\boldsymbol{P}_{\boldsymbol{k}}\boldsymbol{\in}\boldsymbol{P}$*, the approximate filtering needs its execution several times to filter all* $\boldsymbol{t}_{\boldsymbol{k}}$ *indices location and then to store within the classical array*$\boldsymbol{LA}_{\boldsymbol{k}}\left[ \boldsymbol{t}_{\boldsymbol{k}} \right]$***.***

***Trace Note – D3:*** *Below mentioned tracing steps are repeated to find all* $\boldsymbol{t}_{\boldsymbol{k}}$ *exact occurrence of each* $\boldsymbol{P}_{\boldsymbol{k}}\boldsymbol{\in}\boldsymbol{P}$ *at* $\boldsymbol{c}^{\boldsymbol{th}}$ *quantum core* $\boldsymbol{QCore}_{\boldsymbol{c}}$ *by using individual unitary operator* $\boldsymbol{U}_{\boldsymbol{kGetL}}$ *that lets the address registers to access original filtered indices with the help of location register****.*** *So****,*** *further steps are written for* $\boldsymbol{EnQBCEA-MPM}$ *to report all occurrences of pattern*$\boldsymbol{P}_{\boldsymbol{k}}$***.***

1. *Prepare quantum registers of each* $\boldsymbol{c}^{\boldsymbol{th}}$ *quantum core* $\boldsymbol{QCore}_{\boldsymbol{c}}$ *in zero states along with ancilla to set as one****,*** *and store each* $\boldsymbol{P}_{\boldsymbol{k}}\boldsymbol{\in}\boldsymbol{P}$ *of size* $\boldsymbol{w}\boldsymbol{=}\boldsymbol{M}_{\boldsymbol{k}}\boldsymbol{\times}\boldsymbol{log}_{\boldsymbol{2}} \left| \boldsymbol{\Sigma} \right|$ *in register* ${\boldsymbol{|}\boldsymbol{P}\boldsymbol{\rangle}}_{\boldsymbol{DRk}}$

${{\boldsymbol{|}\boldsymbol{\psi}_{\boldsymbol{tq}}\boldsymbol{\rangle}}_{\boldsymbol{k}}}^{\boldsymbol{0}}$**:** ${{\boldsymbol{|}\boldsymbol{T}_{\boldsymbol{0}}\boldsymbol{\rangle}}_{\boldsymbol{QLk}}}^{\boldsymbol{\otimes tq}} {{\boldsymbol{\bigotimes|}\boldsymbol{T}_{\boldsymbol{0}}\boldsymbol{\rangle}}_{\boldsymbol{QAk}}}^{\boldsymbol{\otimes n}} {{\boldsymbol{|}\boldsymbol{T}_{\boldsymbol{[0]}}\boldsymbol{\rangle}}_{\boldsymbol{QDk}}}^{\boldsymbol{\otimes w}}\boldsymbol{\bigotimes}{\boldsymbol{|}\boldsymbol{1}\boldsymbol{\rangle}}_{\boldsymbol{Qk}}$ $\boldsymbol{\bigotimes}{\boldsymbol{|}\boldsymbol{P}\boldsymbol{\rangle}}_{\boldsymbol{DRk}}\boldsymbol{\leftarrow}\left\{ \boldsymbol{P}_{\boldsymbol{1}}\boldsymbol{,.,}\boldsymbol{P}_{\boldsymbol{k}}\boldsymbol{,}\boldsymbol{.,}\boldsymbol{P}_{\boldsymbol{m}} \right\}$ **(32)**

1. *Initialize superposition state in location register and ancilla of* $\boldsymbol{c}^{\boldsymbol{th}}$ *quantum core* $\boldsymbol{QCore}_{\boldsymbol{c}}$ *as*

${{\boldsymbol{|}\boldsymbol{\psi}_{\boldsymbol{tq}}\boldsymbol{\rangle}}_{\boldsymbol{k}}}^{\boldsymbol{1}}$ **:** $\boldsymbol{H}^{\boldsymbol{\otimes(tq+1)}}\left( \boldsymbol{I}^{\boldsymbol{\otimes tq}}\boldsymbol{\bigotimes I} \right) {{\boldsymbol{|}\boldsymbol{\psi}_{\boldsymbol{tq}}\boldsymbol{\rangle}}_{\boldsymbol{k}}}^{\boldsymbol{0}}$

**:** $\frac{\boldsymbol{1}}{\sqrt{\boldsymbol{t}_{\boldsymbol{k}}}}\sum_{\boldsymbol{i=0}}^{\boldsymbol{t}_{\boldsymbol{k}}\boldsymbol{- 1}} {\boldsymbol{|}\boldsymbol{T}_{\boldsymbol{i}}\boldsymbol{\rangle}}_{\boldsymbol{QLk}} {\boldsymbol{\bigotimes|}\boldsymbol{T}_{\boldsymbol{0}}\boldsymbol{\rangle}}_{\boldsymbol{QAk}}\boldsymbol{\bigotimes}{\boldsymbol{|}\boldsymbol{T}_{\boldsymbol{[0]}}\boldsymbol{\rangle}}_{\boldsymbol{QDk}} \boldsymbol{\bigotimes}\left( \frac{\boldsymbol{1}}{\sqrt{\boldsymbol{2}}}\left( \boldsymbol{|0}\boldsymbol{\rangle-}\boldsymbol{|1}\boldsymbol{\rangle} \right) \right)_{\boldsymbol{Qk}}\boldsymbol{\bigotimes}{\boldsymbol{|}\boldsymbol{P}\boldsymbol{\rangle}}_{\boldsymbol{DRk}}$

*Now, data and pattern register used at* $\boldsymbol{c}^{\boldsymbol{th}}$ *quantum core* $\boldsymbol{QCore}_{\boldsymbol{c}}$ *is taking alongside for the further comparison, and separate the ancilla qubit, so we rewrite this state* ${{\boldsymbol{|}\boldsymbol{\psi}_{\boldsymbol{tq}}\boldsymbol{\rangle}}_{\boldsymbol{k}}}^{\boldsymbol{1}}$ *as*

**:** $\frac{\boldsymbol{1}}{\sqrt{\boldsymbol{t}_{\boldsymbol{k}}}} \sum_{\boldsymbol{i=0}}^{\boldsymbol{t}_{\boldsymbol{k}}\boldsymbol{- 1}} {\boldsymbol{|}\boldsymbol{T}_{\boldsymbol{i}}\boldsymbol{\rangle}}_{\boldsymbol{QLk}} {\boldsymbol{\bigotimes|}\boldsymbol{T}_{\boldsymbol{0}}\boldsymbol{\rangle}}_{\boldsymbol{QAk}}\boldsymbol{\bigotimes}{\boldsymbol{|}\boldsymbol{T}_{\boldsymbol{[0]}}\boldsymbol{\rangle}}_{\boldsymbol{QDk}} \boldsymbol{\bigotimes}{\boldsymbol{|}\boldsymbol{P}\boldsymbol{\rangle}}_{\boldsymbol{DRk}}\boldsymbol{\bigotimes}\left( \frac{\boldsymbol{1}}{\sqrt{\boldsymbol{2}}}\left( \boldsymbol{|0}\boldsymbol{\rangle-}\boldsymbol{|1}\boldsymbol{\rangle} \right) \right)_{\boldsymbol{Qk}}$

**:** $\frac{\boldsymbol{1}}{\sqrt{\boldsymbol{t}_{\boldsymbol{k}}}} \sum_{\boldsymbol{i=0}}^{\boldsymbol{t}_{\boldsymbol{k}}\boldsymbol{- 1}} {\boldsymbol{|}\boldsymbol{T}_{\boldsymbol{i}}\boldsymbol{\rangle}}_{\boldsymbol{QLk}} {\boldsymbol{\bigotimes|}\boldsymbol{T}_{\boldsymbol{0}}\boldsymbol{\rangle}}_{\boldsymbol{QAk}}\boldsymbol{\bigotimes}{\boldsymbol{|}\boldsymbol{T}_{\boldsymbol{[0]}}\boldsymbol{\rangle}}_{\boldsymbol{QDk}} \boldsymbol{\bigotimes}{\boldsymbol{|}\boldsymbol{P}\boldsymbol{\rangle}}_{\boldsymbol{DRk}}\boldsymbol{\bigotimes}{\boldsymbol{|}\boldsymbol{-}\boldsymbol{\rangle}}_{\boldsymbol{Qk}}$ **(33)**

1. *Apply an implicit unitary operator* $\boldsymbol{U}_{\boldsymbol{kGetL}}$ *to obtain the original filtered text index as*

${{\boldsymbol{|}\boldsymbol{\psi}_{\boldsymbol{tq}}\boldsymbol{\rangle}}_{\boldsymbol{k}}}^{\boldsymbol{2}}$ **:** $\boldsymbol{U}_{\boldsymbol{kGetL}}\left( {{\boldsymbol{|}\boldsymbol{\psi}_{\boldsymbol{tq}}\boldsymbol{\rangle}}_{\boldsymbol{k}}}^{\boldsymbol{1}} \right)$

$\boldsymbol{U}_{\boldsymbol{kGetL}}$**:** $\boldsymbol{f}\left( {\boldsymbol{|}\boldsymbol{T}_{\boldsymbol{iLk}}\boldsymbol{\rangle}}_{\boldsymbol{QLk}} \right)\boldsymbol{=}\left\{ \boldsymbol{Gets}{\boldsymbol{|}\boldsymbol{T}_{\boldsymbol{iLk}}\boldsymbol{\rangle}}_{\boldsymbol{QAk}} \boldsymbol{as i}^{\boldsymbol{th}}\boldsymbol{Loc as} \right.{\boldsymbol{|}\boldsymbol{T}_{{\boldsymbol{|}\boldsymbol{T}_{\boldsymbol{[i]}}\boldsymbol{\rangle}}_{\boldsymbol{QLk}}}\boldsymbol{\rangle}}_{\boldsymbol{QAk}}\boldsymbol{\leftarrow}{\boldsymbol{|}\boldsymbol{T}_{\boldsymbol{[i]}}\boldsymbol{\rangle}}_{\boldsymbol{QLk}}\boldsymbol{=}\boldsymbol{iLk}$

**:** $\boldsymbol{U}_{\boldsymbol{GetL}}\left( \frac{\boldsymbol{1}}{\sqrt{\boldsymbol{t}_{\boldsymbol{k}}}} \sum_{\boldsymbol{i=0}}^{\boldsymbol{t}_{\boldsymbol{k}}\boldsymbol{- 1}} {{\boldsymbol{|}\boldsymbol{T}_{\boldsymbol{i}}\boldsymbol{\rangle}}_{\boldsymbol{QLk}}\boldsymbol{\bigotimes|}\boldsymbol{T}_{{\boldsymbol{|}\boldsymbol{T}_{\boldsymbol{[i]}}\boldsymbol{\rangle}}_{\boldsymbol{QLk}}}\boldsymbol{\rangle}}_{\boldsymbol{QAk}} \boldsymbol{\bigotimes}{\boldsymbol{|}\boldsymbol{T}_{\boldsymbol{[0]}}\boldsymbol{\rangle}}_{\boldsymbol{QDk}} \boldsymbol{\bigotimes}{\boldsymbol{|}\boldsymbol{P}\boldsymbol{\rangle}}_{\boldsymbol{DRk}}\boldsymbol{\bigotimes}{\boldsymbol{|}\boldsymbol{-}\boldsymbol{\rangle}}_{\boldsymbol{Qk}} \right)$

**:** $\frac{\boldsymbol{1}}{\sqrt{\boldsymbol{t}_{\boldsymbol{k}}}} \sum_{\boldsymbol{i=0}}^{\boldsymbol{t}_{\boldsymbol{k}}\boldsymbol{- 1}} {{\boldsymbol{|}\boldsymbol{T}_{\boldsymbol{i}}\boldsymbol{\rangle}}_{\boldsymbol{QLk}}\boldsymbol{\bigotimes|}\boldsymbol{T}_{\boldsymbol{iLk}}\boldsymbol{\rangle}}_{\boldsymbol{QAk}} \boldsymbol{\bigotimes}{\boldsymbol{|}\boldsymbol{T}_{\boldsymbol{[0]}}\boldsymbol{\rangle}}_{\boldsymbol{QDk}} \boldsymbol{\bigotimes}{\boldsymbol{|}\boldsymbol{P}\boldsymbol{\rangle}}_{\boldsymbol{DRk}}\boldsymbol{\bigotimes}{\boldsymbol{|}\boldsymbol{-}\boldsymbol{\rangle}}_{\boldsymbol{Qk}}$ **(34)**

1. *For all* ${\boldsymbol{|}\boldsymbol{T}_{\boldsymbol{iLk}}\boldsymbol{\rangle}}_{\boldsymbol{QAk}}$ *in their separate uniform quantum superposition state* ${{\boldsymbol{|}\boldsymbol{\psi}_{\boldsymbol{tq}}\boldsymbol{\rangle}}_{\boldsymbol{k}}}^{\boldsymbol{2}}$***,*** *load data at* ${\boldsymbol{|}\boldsymbol{T}_{\boldsymbol{[iLk]}}\boldsymbol{\rangle}}_{\boldsymbol{QDk}}$ *as per entangled* ${\boldsymbol{|}\boldsymbol{T}_{\boldsymbol{iLk}}\boldsymbol{\rangle}}_{\boldsymbol{QAk}}$ *by applying* $\boldsymbol{QMEM Transformation}$ *of Eq. (1)*

$$\boldsymbol{QMEM Transformation}\boldsymbol{\leftarrow}\left( \boldsymbol{U}_{\boldsymbol{QMEM}}\boldsymbol{\leftarrow}\left( {\boldsymbol{U}^{\boldsymbol{\dagger}}}_{\boldsymbol{Swap}}\left( \boldsymbol{U}_{\boldsymbol{Load}} \left( \boldsymbol{U}_{\boldsymbol{Swap}} \right) \right) \right) \right)$$

*Unitary* $\boldsymbol{U}_{\boldsymbol{Load}}$ *of Eq. (3) is directly applied for data transformation from memory to register*

${{\boldsymbol{|}\boldsymbol{\psi}_{\boldsymbol{tq}}\boldsymbol{\rangle}}_{\boldsymbol{k}}}^{\boldsymbol{3}}$ **:** $\boldsymbol{U}_{\boldsymbol{Load}}\left( {{\boldsymbol{|}\boldsymbol{\psi}_{\boldsymbol{tq}}\boldsymbol{\rangle}}_{\boldsymbol{k}}}^{\boldsymbol{2}} \right)$

**:**$\boldsymbol{U}_{\boldsymbol{Load}}\left( {\boldsymbol{|}\boldsymbol{T}_{\boldsymbol{iLk}}\boldsymbol{\rangle}}_{\boldsymbol{QAk}}\boldsymbol{\bigotimes}{\boldsymbol{|}\boldsymbol{T}_{\boldsymbol{[0]}}\boldsymbol{\rangle}}_{\boldsymbol{QDk}} \right)\boldsymbol{=}\boldsymbol{U}_{\boldsymbol{Load}}\left( {\boldsymbol{|}\boldsymbol{T}_{\boldsymbol{iLk}}\boldsymbol{\rangle}}_{\boldsymbol{QAk}}\boldsymbol{\bigotimes}{\boldsymbol{|}\boldsymbol{T}_{\left[ \boldsymbol{0}\bigoplus\boldsymbol{iLk} \right]}\boldsymbol{\rangle}}_{\boldsymbol{QDk}} \right)$

**:** $\frac{\boldsymbol{1}}{\sqrt{\boldsymbol{t}_{\boldsymbol{k}}}} \sum_{\boldsymbol{iLk=0}}^{\boldsymbol{t}_{\boldsymbol{k}}\boldsymbol{- 1}} {\boldsymbol{|}\boldsymbol{T}_{\boldsymbol{iLk}}\boldsymbol{\rangle}}_{\boldsymbol{QAk}} \boldsymbol{\bigotimes}{\boldsymbol{|}\boldsymbol{T}_{\boldsymbol{[iLk]}}\boldsymbol{\rangle}}_{\boldsymbol{QDk}} \boldsymbol{\bigotimes}{\boldsymbol{|}\boldsymbol{P}\boldsymbol{\rangle}}_{\boldsymbol{DRk}}\boldsymbol{\bigotimes}{\boldsymbol{|}\boldsymbol{-}\boldsymbol{\rangle}}_{\boldsymbol{Qk}}$ **(35)**

*Repeat* $\boldsymbol{GSO}$ *of Eq. (6) for* $\boldsymbol{O}\left( \sqrt{{\boldsymbol{t}_{\boldsymbol{k}}}/{{\boldsymbol{t}_{\boldsymbol{k}}}^{\boldsymbol{'}}}} \right)$ *times in superposition* ${{\boldsymbol{|}\boldsymbol{\psi}_{\boldsymbol{tq}}\boldsymbol{\rangle}}_{\boldsymbol{k}}}^{\boldsymbol{3}}$ *by* $\boldsymbol{QEM Operation}$ *that is implicitly applied through* $\boldsymbol{U}_{\boldsymbol{kComp}}$ *for exact matching of* $\boldsymbol{M}_{\boldsymbol{k}}\boldsymbol{\times}\boldsymbol{log}_{\boldsymbol{2}} \left| \boldsymbol{\Sigma} \right|$ *qubits size as*

$\boldsymbol{GSO Opeartion\leftarrow}\left( {\boldsymbol{D\leftarrow}\boldsymbol{U}}_{\boldsymbol{Diff}}\left( \boldsymbol{O\leftarrow}\left( \boldsymbol{U}_{\boldsymbol{Mark}}\left( \boldsymbol{U}_{\boldsymbol{Comp}} \right) \right) \right) \right)$

*Apply* $\boldsymbol{QEM Operation}$ *by* $\boldsymbol{U}_{\boldsymbol{kComp}}$ *of Eq. (5) to find index* ${\boldsymbol{|}\boldsymbol{T}_{\boldsymbol{iLk}}\boldsymbol{\rangle}}_{\boldsymbol{QAk}}$ *to assure exact match*

${{\boldsymbol{|}\boldsymbol{\psi}_{\boldsymbol{tq}}\boldsymbol{\rangle}}_{\boldsymbol{k}}}^{\boldsymbol{3}}$ **:** $\boldsymbol{U}_{\boldsymbol{kComp}}\left( {{\boldsymbol{|}\boldsymbol{\psi}_{\boldsymbol{tq}}\boldsymbol{\rangle}}_{\boldsymbol{k}}}^{\boldsymbol{3}} \right)$

**:** $\boldsymbol{f}\left( {\boldsymbol{|}\boldsymbol{T}_{\boldsymbol{iLk}}\boldsymbol{\rangle}}_{\boldsymbol{QAk}} \right)\boldsymbol{=}\left\{ \begin{aligned} \begin{matrix} \boldsymbol{0,} & \boldsymbol{if} \end{matrix}{\boldsymbol{|}\boldsymbol{T}_{\boldsymbol{[iLk to iLk+}\boldsymbol{M}_{\boldsymbol{k}}\boldsymbol{-1]}}\boldsymbol{\rangle}}_{\boldsymbol{QDk}}\boldsymbol{\neq}{\boldsymbol{|}\boldsymbol{P}_{\left[ \boldsymbol{0 to}\boldsymbol{M}_{\boldsymbol{k}}\boldsymbol{-1} \right]}\boldsymbol{\rangle}}_{\boldsymbol{DRk}} \\ \begin{matrix} \boldsymbol{1,} & \boldsymbol{if} \end{matrix}{\boldsymbol{|}\boldsymbol{T}_{\boldsymbol{[iLk to iLk+}\boldsymbol{M}_{\boldsymbol{k}}\boldsymbol{-1]}}\boldsymbol{\rangle}}_{\boldsymbol{QDk}}\boldsymbol{=}{\boldsymbol{|}\boldsymbol{P}_{\left[ \boldsymbol{0 to}\boldsymbol{M}_{\boldsymbol{k}}\boldsymbol{-1} \right]}\boldsymbol{\rangle}}_{\boldsymbol{DRk}} \end{aligned} \right.$ **(36)**

$\boldsymbol{if}\left( \boldsymbol{f}\left( {\boldsymbol{|}\boldsymbol{T}_{\boldsymbol{iLk}}\boldsymbol{\rangle}}_{\boldsymbol{QAk}} \right)\boldsymbol{= =1} \right)\boldsymbol{then}$

*Apply unitary* $\boldsymbol{U}_{\boldsymbol{Mark}}$ *of Eq. (7) to mark* ${\boldsymbol{|}\boldsymbol{T}_{\boldsymbol{iLk}}\boldsymbol{\rangle}}_{\boldsymbol{QAk}}$ *as* $\left( \boldsymbol{I-}\boldsymbol{2}{\boldsymbol{|}\boldsymbol{T}_{\boldsymbol{i}}\boldsymbol{\rangle}}_{\boldsymbol{QA}}{\boldsymbol{\langle}\boldsymbol{T}_{\boldsymbol{i}}\boldsymbol{|}}_{\boldsymbol{QA}} \right)\boldsymbol{\bigotimes}\left( {\boldsymbol{|}\boldsymbol{T}_{\boldsymbol{i}}\boldsymbol{\rangle}}_{\boldsymbol{QA}}\boldsymbol{\bigotimes|q\rangle} \right)$

${{\boldsymbol{|}\boldsymbol{\psi}_{\boldsymbol{tq}}\boldsymbol{\rangle}}_{\boldsymbol{k}}}^{\boldsymbol{4}}$ **:** $\boldsymbol{U}_{\boldsymbol{Mark}}\left( {{\boldsymbol{|}\boldsymbol{\psi}_{\boldsymbol{tq}}\boldsymbol{\rangle}}_{\boldsymbol{k}}}^{\boldsymbol{3}} \right)$

**:** $\boldsymbol{U}_{\boldsymbol{Mark}}\left( \frac{\boldsymbol{1}}{\sqrt{\boldsymbol{t}_{\boldsymbol{k}}}} \sum_{\boldsymbol{iLk=0}}^{\boldsymbol{t}_{\boldsymbol{k}}\boldsymbol{- 1}} {\boldsymbol{|}\boldsymbol{T}_{\boldsymbol{iLk}}\boldsymbol{\rangle}}_{\boldsymbol{QAk}} \boldsymbol{\bigotimes}{\boldsymbol{|}\boldsymbol{T}_{\boldsymbol{[iLk]}}\boldsymbol{\rangle}}_{\boldsymbol{QDk}} \boldsymbol{\bigotimes}{\boldsymbol{|}\boldsymbol{P}\boldsymbol{\rangle}}_{\boldsymbol{DRk}}\boldsymbol{\bigotimes}{\boldsymbol{|}\boldsymbol{-}\boldsymbol{\rangle}}_{\boldsymbol{Qk}} \right)$

**:** $\boldsymbol{U}_{\boldsymbol{Mark}}$ *inverts* ${\boldsymbol{|}\boldsymbol{T}_{\boldsymbol{iLk}}\boldsymbol{\rangle}}_{\boldsymbol{QAk}}$ *to* $\boldsymbol{-}{\boldsymbol{|}\boldsymbol{T}_{\boldsymbol{iLk}}\boldsymbol{\rangle}}_{\boldsymbol{QAk}}$ *if solution exist, otherwise* $\sum_{\begin{aligned} \boldsymbol{j \in}\boldsymbol{t}_{\boldsymbol{k}} \\ \boldsymbol{j \neq iLk} \end{aligned}} {\boldsymbol{|}\boldsymbol{T}_{\boldsymbol{j}}\boldsymbol{\rangle}}_{\boldsymbol{QAk}}$

**:** $\left\{ \begin{aligned} \left( \boldsymbol{I-}\boldsymbol{2}{\boldsymbol{|}\boldsymbol{T}_{\boldsymbol{iLk}}\boldsymbol{\rangle}}_{\boldsymbol{QAk}}{\boldsymbol{\langle}\boldsymbol{T}_{\boldsymbol{iLk}}\boldsymbol{|}}_{\boldsymbol{QAk}} \right){\boldsymbol{|}\boldsymbol{T}_{\boldsymbol{iLk}}\boldsymbol{\rangle}}_{\boldsymbol{QAk}}\boldsymbol{=}{\boldsymbol{|}\boldsymbol{T}_{\boldsymbol{iLk}}\boldsymbol{\rangle}}_{\boldsymbol{QAk}}\boldsymbol{-2}{\boldsymbol{|}\boldsymbol{T}_{\boldsymbol{iLk}}\boldsymbol{\rangle}}_{\boldsymbol{QAk}}\left\langle\boldsymbol{T}_{\boldsymbol{iLk}} | \boldsymbol{T}_{\boldsymbol{iLk}} \right\rangle_{\boldsymbol{QAk}}\boldsymbol{=-}{\boldsymbol{|}\boldsymbol{T}_{\boldsymbol{iLk}}\boldsymbol{\rangle}}_{\boldsymbol{QAk}} \\ \left( \boldsymbol{I-}\boldsymbol{2}{\boldsymbol{|}\boldsymbol{T}_{\boldsymbol{iLk}}\boldsymbol{\rangle}}_{\boldsymbol{QAk}}{\boldsymbol{\langle}\boldsymbol{T}_{\boldsymbol{iLk}}\boldsymbol{|}}_{\boldsymbol{QAk}} \right){\boldsymbol{|}\boldsymbol{T}_{\boldsymbol{j}}\boldsymbol{\rangle}}_{\boldsymbol{QAk}}\boldsymbol{=}{\boldsymbol{|}\boldsymbol{T}_{\boldsymbol{iLk}}\boldsymbol{\rangle}}_{\boldsymbol{QAk}}\boldsymbol{-2}{\boldsymbol{|}\boldsymbol{T}_{\boldsymbol{iLk}}\boldsymbol{\rangle}}_{\boldsymbol{QAk}}\left\langle\boldsymbol{T}_{\boldsymbol{iLk}} | \boldsymbol{T}_{\boldsymbol{j}} \right\rangle_{\boldsymbol{QAk}}\boldsymbol{=}{\boldsymbol{|}\boldsymbol{T}_{\boldsymbol{iLk}}\boldsymbol{\rangle}}_{\boldsymbol{QAk}} \end{aligned} \right.$

**:** $\frac{\boldsymbol{1}}{\sqrt{\boldsymbol{t}_{\boldsymbol{k}}}} \sum_{\boldsymbol{iL=0}}^{\boldsymbol{t}_{\boldsymbol{k}}\boldsymbol{- 1}} {\boldsymbol{-|}\boldsymbol{T}_{\boldsymbol{iLk}}\boldsymbol{\rangle}}_{\boldsymbol{QAk}} {\boldsymbol{\bigotimes|}\boldsymbol{T}_{\boldsymbol{[iLk]}}\boldsymbol{\rangle}}_{\boldsymbol{QDk}} \boldsymbol{\bigotimes}{\boldsymbol{|}\boldsymbol{P}\boldsymbol{\rangle}}_{\boldsymbol{DRk}}\boldsymbol{\bigotimes}{\boldsymbol{|}\boldsymbol{-}\boldsymbol{\rangle}}_{\boldsymbol{Qk}}$

*Therefore, each oracle call computes negated amplitude for index* ${\boldsymbol{-|}\boldsymbol{T}_{\boldsymbol{iLk}}\boldsymbol{\rangle}}_{\boldsymbol{QAk}}$ *to* *obtain quantum state* ${{\boldsymbol{|}\boldsymbol{\psi}_{\boldsymbol{tq}}\boldsymbol{\rangle}}_{\boldsymbol{k}}}^{\boldsymbol{4}}$ *from the passed state* ${{\boldsymbol{|}\boldsymbol{\psi}_{\boldsymbol{tq}}\boldsymbol{\rangle}}_{\boldsymbol{k}}}^{\boldsymbol{3}}$ *to the oracle*

$\boldsymbol{O}\left( {{\boldsymbol{|}\boldsymbol{\psi}_{\boldsymbol{tq}}\boldsymbol{\rangle}}_{\boldsymbol{k}}}^{\boldsymbol{4}} \right)\boldsymbol{=}\left( \boldsymbol{I-}\boldsymbol{2}{\boldsymbol{|}\boldsymbol{T}_{\boldsymbol{iLk}}\boldsymbol{\rangle}}_{\boldsymbol{QAk}}{\boldsymbol{\langle}\boldsymbol{T}_{\boldsymbol{iLk}}\boldsymbol{|}}_{\boldsymbol{QAk}} \right) {{\boldsymbol{|}\boldsymbol{\psi}_{\boldsymbol{tq}}\boldsymbol{\rangle}}_{\boldsymbol{k}}}^{\boldsymbol{3}}$

$\boldsymbol{=}{{\boldsymbol{|}\boldsymbol{\psi}_{\boldsymbol{tq}}\boldsymbol{\rangle}}_{\boldsymbol{k}}}^{\boldsymbol{3}}\boldsymbol{-}\boldsymbol{2}{\boldsymbol{|}\boldsymbol{T}_{\boldsymbol{iLk}}\boldsymbol{\rangle}}_{\boldsymbol{QAk}}\left\langle\boldsymbol{T}_{\boldsymbol{iLk}} | \boldsymbol{\psi}_{\boldsymbol{tq}} \right\rangle_{\boldsymbol{QAk}}$ ***Where,*** $\left\langle\boldsymbol{T}_{\boldsymbol{iLk}} | \boldsymbol{\psi}_{\boldsymbol{tq}} \right\rangle_{\boldsymbol{QAk}}\boldsymbol{=}\frac{\boldsymbol{1}}{\sqrt{\boldsymbol{t}_{\boldsymbol{k}}}}$

$\boldsymbol{=}{{\boldsymbol{|}\boldsymbol{\psi}_{\boldsymbol{tq}}\boldsymbol{\rangle}}_{\boldsymbol{k}}}^{\boldsymbol{3}}\boldsymbol{-}\frac{\boldsymbol{2}}{\sqrt{\boldsymbol{t}_{\boldsymbol{k}}}}{\boldsymbol{|}\boldsymbol{T}_{\boldsymbol{iLk}}\boldsymbol{\rangle}}_{\boldsymbol{QAk}}$ **(37)**

*Apply diffusion operator*$\boldsymbol{D}\left( \boldsymbol{U}_{\boldsymbol{Diff}}\left( \boldsymbol{O} \right) \right)$ *of Eq. (8) for the inversion about the mean as*

$\boldsymbol{D}\left( \boldsymbol{U}_{\boldsymbol{Diff}}\left( \boldsymbol{O} \right) \right)\boldsymbol{=}\boldsymbol{D}\left( \left( \boldsymbol{2}\boldsymbol{|}\boldsymbol{\psi}_{\boldsymbol{n}}\boldsymbol{\rangle}\boldsymbol{\langle}\boldsymbol{\psi}_{\boldsymbol{n}}\boldsymbol{|}\boldsymbol{-I} \right)\left( \boldsymbol{O} \right) \right)\boldsymbol{=D}\left( \boldsymbol{H}^{\boldsymbol{\otimes n}}\left( \boldsymbol{2}\boldsymbol{|}\boldsymbol{0}\boldsymbol{\rangle}\boldsymbol{\langle}\boldsymbol{0}\boldsymbol{|}\boldsymbol{-I} \right)\boldsymbol{H}^{\boldsymbol{\otimes n}}\left( \boldsymbol{O} \right) \right)$

${{\boldsymbol{|}\boldsymbol{\psi}_{\boldsymbol{tq}}\boldsymbol{\rangle}}_{\boldsymbol{k}}}^{\boldsymbol{5}}$ **:** $\boldsymbol{U}_{\boldsymbol{Diff}}\left( {{\boldsymbol{|}\boldsymbol{\psi}_{\boldsymbol{tq}}\boldsymbol{\rangle}}_{\boldsymbol{k}}}^{\boldsymbol{4}} \right)$

**:** $\left( \boldsymbol{2}{{\boldsymbol{|}\boldsymbol{\psi}_{\boldsymbol{tq}}\boldsymbol{\rangle}}_{\boldsymbol{k}}}^{\boldsymbol{4}}{{\boldsymbol{\langle}\boldsymbol{\psi}_{\boldsymbol{tq}}\boldsymbol{|}}_{\boldsymbol{k}}}^{\boldsymbol{4}}\boldsymbol{-I} \right)\left( {{\boldsymbol{|}\boldsymbol{\psi}_{\boldsymbol{tq}}\boldsymbol{\rangle}}_{\boldsymbol{k}}}^{\boldsymbol{4}}\boldsymbol{-}\frac{\boldsymbol{2}}{\sqrt{\boldsymbol{t}_{\boldsymbol{k}}}}{\boldsymbol{|}\boldsymbol{T}_{\boldsymbol{iLk}}\boldsymbol{\rangle}}_{\boldsymbol{QAk}} \right)$

**:** $\boldsymbol{2}{{\boldsymbol{|}\boldsymbol{\psi}_{\boldsymbol{tq}}\boldsymbol{\rangle}}_{\boldsymbol{k}}}^{\boldsymbol{4}}\boldsymbol{-}{{\boldsymbol{|}\boldsymbol{\psi}_{\boldsymbol{tq}}\boldsymbol{\rangle}}_{\boldsymbol{k}}}^{\boldsymbol{4}}\boldsymbol{-}\frac{\boldsymbol{2}^{\boldsymbol{2}}}{\boldsymbol{t}_{\boldsymbol{k}}}{{\boldsymbol{|}\boldsymbol{\psi}_{\boldsymbol{tq}}\boldsymbol{\rangle}}_{\boldsymbol{k}}}^{\boldsymbol{4}}\boldsymbol{+}\frac{\boldsymbol{2}}{\sqrt{\boldsymbol{t}_{\boldsymbol{k}}}} {\boldsymbol{|}\boldsymbol{T}_{\boldsymbol{iLk}}\boldsymbol{\rangle}}_{\boldsymbol{QAk}}$

*Therefore, after each diffusion operation, the amplitude of marked index*${\boldsymbol{|}\boldsymbol{T}_{\boldsymbol{iLk}}\boldsymbol{\rangle}}_{\boldsymbol{QAk}}$ *increases by roughly* $\left( \boldsymbol{+}\frac{\boldsymbol{2}}{\sqrt{\boldsymbol{t}_{\boldsymbol{k}}}} \right)$ *and finally on passing* ${{\boldsymbol{|}\boldsymbol{\psi}_{\boldsymbol{tq}}\boldsymbol{\rangle}}_{\boldsymbol{k}}}^{\boldsymbol{4}}$ *to diffusion, we get state* ${{\boldsymbol{|}\boldsymbol{\psi}_{\boldsymbol{tq}}\boldsymbol{\rangle}}_{\boldsymbol{k}}}^{\boldsymbol{5}}$ *as*

${{\boldsymbol{|}\boldsymbol{\psi}_{\boldsymbol{tq}}\boldsymbol{\rangle}}_{\boldsymbol{k}}}^{\boldsymbol{5}}$ **:** $\boldsymbol{D}\left( {{\boldsymbol{|}\boldsymbol{\psi}_{\boldsymbol{tq}}\boldsymbol{\rangle}}_{\boldsymbol{k}}}^{\boldsymbol{4}} \right)\boldsymbol{=}{{\boldsymbol{|}\boldsymbol{\psi}_{\boldsymbol{tq}}\boldsymbol{\rangle}}_{\boldsymbol{k}}}^{\boldsymbol{4}}\boldsymbol{-}\frac{\boldsymbol{2}^{\boldsymbol{2}}}{\boldsymbol{t}_{\boldsymbol{k}}}{{\boldsymbol{|}\boldsymbol{\psi}_{\boldsymbol{tq}}\boldsymbol{\rangle}}_{\boldsymbol{k}}}^{\boldsymbol{4}}\boldsymbol{+}\frac{\boldsymbol{2}}{\sqrt{\boldsymbol{t}_{\boldsymbol{k}}}} {\boldsymbol{|}\boldsymbol{T}_{\boldsymbol{iLk}}\boldsymbol{\rangle}}_{\boldsymbol{QAk}}$ **(38)**

***Trace Note – D4:*** *The operator* $\boldsymbol{GSO}$ *is repeated until required* $\left( \boldsymbol{\pi}/\boldsymbol{4}\sqrt{{\boldsymbol{t}_{\boldsymbol{k}}}/{{\boldsymbol{t}_{\boldsymbol{k}}}^{\boldsymbol{'}}}} \right)$ *number of iterations are not completed;* *thus, this assures the final quantum state that must be containing a high probable solution at marked index*${\boldsymbol{|}\boldsymbol{T}_{\boldsymbol{iLk}}\boldsymbol{\rangle}}_{\boldsymbol{QAk}}$ *corresponding to*$\boldsymbol{P}_{\boldsymbol{k}}\boldsymbol{\in}\boldsymbol{P}$***.***

1. *Measure final state after the needed iterations as* ${{\boldsymbol{|}\boldsymbol{\psi}_{\boldsymbol{tq}}\boldsymbol{\rangle}}_{\boldsymbol{k}}}^{\left( \boldsymbol{\pi}/\boldsymbol{4}\sqrt{{\boldsymbol{t}_{\boldsymbol{k}}}/{{\boldsymbol{t}_{\boldsymbol{k}}}^{\boldsymbol{'}}}} \right)}$ *to get the desired index* ${\boldsymbol{|}\boldsymbol{T}_{\boldsymbol{iLk}}\boldsymbol{\rangle}}_{\boldsymbol{QAk}}$ *as high probable solution.*

***Trace Note – D5:*** *The quantum state gets collapsed after each measurement****,*** *so its repetition will ensure to report all* ${\boldsymbol{t}_{\boldsymbol{k}}}^{\boldsymbol{'}}$ *index location* *of* $\boldsymbol{P}_{\boldsymbol{k}}\boldsymbol{\in}\boldsymbol{P}$ *identified by the* $\boldsymbol{c}^{\boldsymbol{th}}$ *quantum core* $\boldsymbol{QCore}_{\boldsymbol{c}}$ *as pattern match over likely* $\boldsymbol{t}_{\boldsymbol{k}}$ *filtered indices stored in*$\boldsymbol{LA}_{\boldsymbol{k}}\left[ \boldsymbol{t}_{\boldsymbol{k}} \right]$***.*** *Still, the output based on behalf of* $\boldsymbol{k}^{\boldsymbol{th}}$ *exact search unitary* $\boldsymbol{U}_{\boldsymbol{kComp}}$ *is used within the*$\boldsymbol{GSO}$ *operator****.***

1. *Verify for* $\boldsymbol{k}^{\boldsymbol{th}}$ *pattern using address register* *of* $\boldsymbol{c}^{\boldsymbol{th}}$ *core* $\boldsymbol{QCore}_{\boldsymbol{c}}$ *to report the pattern occurrence at index*${\boldsymbol{|}\boldsymbol{T}_{\boldsymbol{iLk}}\boldsymbol{\rangle}}_{\boldsymbol{QAk}}$ *as* ${\boldsymbol{|}\boldsymbol{T}_{\boldsymbol{[iLk to iLk+}\boldsymbol{M}_{\boldsymbol{k}}\boldsymbol{-1]}}\boldsymbol{\rangle}}_{\boldsymbol{QDk}}\boldsymbol{= =}{\boldsymbol{|}\boldsymbol{P}_{\left[ \boldsymbol{0 to}\boldsymbol{M}_{\boldsymbol{k}}\boldsymbol{-1} \right]}\boldsymbol{\rangle}}_{\boldsymbol{DRk}}$ **(39)**
